# Neonatal mortality and morbidity in Down syndrome in the time of prenatal aneuploidy testing: a retrospective cohort study

**DOI:** 10.1007/s00431-022-04686-3

**Published:** 2022-11-09

**Authors:** Maurike Dorothea de Groot-van der Mooren, Brigitta Catharina Scheerman, Lukas Augustinus Johannes Rammeloo, Hester van Wieringen, Anne-Marie van Wermeskerken, Roos van der Plas, Peter de Winter, Michel Emile Weijerman, Martina Cornelia Cornel, Anton Hubertus van Kaam

**Affiliations:** 1 Department of Neonatology, Amsterdam UMC Location University of Amsterdam, Meibergdreef 9, Amsterdam, the Netherlands; 2 Amsterdam Public Health Research Institute, Amsterdam, the Netherlands; 3 Amsterdam Reproduction & Development Research Institute, Amsterdam, the Netherlands; 4 Department of Pediatrics, Amsterdam UMC Location University of Amsterdam, Meibergdreef 9, Amsterdam, the Netherlands; 5 Department of Pediatric Cardiology, Amsterdam UMC Location University of Amsterdam, Meibergdreef 9, Amsterdam, the Netherlands; 6 Department of Pediatrics, St. Antonius Ziekenhuis, Koekoekslaan 1, Nieuwegein, the Netherlands; 7 Department of Pediatrics, Flevoziekenhuis, Hospitaalweg 1, Almere, the Netherlands; 8 Department of Pediatrics, Spaarne Gasthuis, Boerhaavelaan 22, Haarlem & Spaarnepoort 1, Hoofddorp, the Netherlands; 9 Department of Development and Regeneration, KU Leuven, Leuven, Belgium; 10 Child & Youth Institute, KU Leuven, Leuven, Belgium; 11 Department of Pediatrics, Alrijne Hospital, Simon, Smitweg 1, Leiderdorp, the Netherlands; 12 Department of Human Genetics, Amsterdam UMC Location Vrije Universiteit Amsterdam, De Boelelaan 1117, Amsterdam, the Netherlands

**Keywords:** Down syndrome, Neonatal mortality, Neonatal morbidity, Congenital defects, Persistent pulmonary hypertension of the neonate, Prenatal aneuploidy screening

## Abstract

The total uptake of prenatal aneuploidy screening for Down syndrome (DS) is increasing worldwide. As a result of increasing prenatal diagnosis of DS and subsequent termination of pregnancy, livebirth prevalence of DS is decreasing. The aim of this study is to explore the impact of an increasing uptake of prenatal aneuploidy screening on the neonatal mortality and morbidity in DS. This is a retrospective cohort study of 253 neonates with DS born between 2012 and 2018 that were seen at the outpatient clinic of five hospitals in the Netherlands. The medical files were reviewed for maternal and neonatal characteristics and neonatal morbidities. The Dutch national birth registry (Perined) provided mortality numbers of neonates with DS. The results were interpreted in the context of other published studies. Neonatal mortality in DS remained stable, ranging from 1.4 to 3.6%. A congenital heart defect (CHD) was found in 138 of the 251 neonates (55.0%) with atrial septal defect, atrioventricular septal defect, and ventricular septal defect being the most common. The type of CHD in DS did not change over time. Gastro-intestinal defects were present in 22 of the 252 neonates with DS (8.7%), with duodenal atresia as the most reported anomaly. Persistent pulmonary hypertension of the neonate (PPHN) was found in 31 of the 251 infants (12.4%).

*  Conclusions*: Although uptake of prenatal aneuploidy screening increased, neonatal mortality and morbidity in DS appears to be stable. An increased incidence of PPHN was found.

**Table:** 

**What is Known:** *• The total uptake of prenatal aneuploidy screening for Down syndrome is increasing worldwide.* *• As a result of increasing prenatal diagnosis of Down syndrome and subsequent termination of pregnancy, the livebirth prevalence of Down syndrome is decreasing.*	
**What is New:** *• Although uptake of prenatal aneuploidy screening increased, neonatal mortality and morbidity in Down syndrome appears to be stable.* *• An increased incidence of persistent pulmonary hypertension of the neonate was found.*

**What is Known:**

*• The total uptake of prenatal aneuploidy screening for Down syndrome is increasing worldwide.*

*• As a result of increasing prenatal diagnosis of Down syndrome and subsequent termination of pregnancy, the livebirth prevalence of Down syndrome is decreasing.*

**What is New:**

*• Although uptake of prenatal aneuploidy screening increased, neonatal mortality and morbidity in Down syndrome appears to be stable.*

*• An increased incidence of persistent pulmonary hypertension of the neonate was found.*

## Introduction

Down syndrome (DS), or trisomy 21, is a genetic condition that is characterized by a variety of dysmorphic features and congenital malformations, including congenital heart disease (CHD) and gastro-intestinal disease. In addition, DS is associated with several impairments, such as hypothyroidism, celiac disease, diabetes mellitus, and developmental delay [1]. Over the last decades, the survival rate of children with DS has increased due to early surgical treatment of CHD and an improved treatment of gastro-intestinal malformations [2]. Prenatal aneuploidy screening allows for early detection of a foetus with DS [3]. In the Netherlands, a national screening program was implemented in 2007 consisting of the so-called first-trimester combined screening test (FCT). The risk assessment for DS with FCT is based on maternal age, foetal nuchal translucency thickness, maternal serum-free β-human chorionic gonadotrophin concentrations, and pregnancy-associated plasma protein-A. Furthermore, a structural foetal anomaly scan in the second trimester was offered to all pregnant women. Non-invasive prenatal testing (NIPT) was introduced in 2014 to women with an increased risk for trisomy 13, 18 and 21, based on the results of the FCT or their medical history. In 2017, NIPT became available to all pregnant women as a first-tier test. NIPT is a highly accurate screening test based on an analysis of cell-free DNA that circulates in the mother's blood and can be used from 10 weeks gestational age. However, NIPT is not diagnostic, and confirmation of a positive result by invasive testing (chorionic villus sampling or amniocentesis) is recommended [4].

All Dutch women are offered information on prenatal screening for foetal abnormalities by their obstetric care provider, generally a midwife. Women expressing an interest in this information are offered a 30-min counselling session by a certified obstetric-care professional. Since 2017, women are counselled for both FCT and NIPT, as well as the 20-week foetal anomaly ultrasound scan. Women can choose to opt in or out for either or both screening programs. During counselling women are given information regarding NIPT versus FCT, the common trisomies, the meaning of possible test-results and diagnostic follow-up testing.

The total uptake of prenatal aneuploidy screening for DS (FCT and NIPT) increased in the Netherlands from 14.8% in 2007 to 45.9% in 2018 [5]. The aim of prenatal aneuploidy screening is to promote reproductive autonomy, which means that in the case of an abnormal test-result, expectant parents can prepare for the birth of a child with DS or decide to terminate the pregnancy. With DS-related elective terminations increasing, reduction in overall DS livebirths is observed in almost all countries. This includes the Netherlands, where DS livebirth prevalence decreased gradually since 2002 (15.9 per 10 000) until 2018 (9.9 per 10 000) [6].

The impact of an increasing uptake of prenatal aneuploidy screening on neonatal mortality and morbidity in DS is not well known. Co-occurrence of congenital defects may influence the parents’ decision to terminate the pregnancy in the case of DS [7]. In addition, some defects like an atrioventricular septal defect or a small bowel obstruction are strongly associated with DS and more frequently identified through a foetal anomaly scan [8]. This may lead to further prenatal aneuploidy testing for DS, also when women initially did not opt for prenatal aneuploidy testing. As a result, subsequent selective abortion of foetuses with DS and certain congenital defects may occur. If these congenital defects are also associated with a worse outcome, neonatal mortality will decrease.

Therefore, the aim of this study is to explore the impact of an increasing uptake of prenatal aneuploidy screening on the neonatal mortality and morbidity in DS. We hypothesized that neonatal mortality is on the decrease and a more favourable phenotype in live born neonates with DS is currently present compared to the era before a prenatal aneuploidy screening program was introduced.

## Materials and methods

### Study design

This retrospective cohort study comprised 253 neonates with DS born between 2012 and 2018 and seen at the outpatient clinic of five hospitals in the Netherlands—one academic hospital and four surrounding general hospitals. In the Netherlands, all neonates with DS will have out-patient follow-up visits at the hospital nearby their residence. If prenatal ultrasound findings (such as a CHD) results in a birth in an academic hospital, the follow-up of these patients with DS is done in the general hospital nearby their residence. Furthermore, the academic and regional hospitals included are in the same area. Therefore, we would like to describe our cohort as a group neonates with DS that were born in 2012–2018 living in a particular region in the Netherlands, representing a service area of circa 5 million people compared with approximately 17 million citizens in the Netherlands. The number of children that were born in 2012–2018 and seen at these outpatient clinics determined the sample size.

Only neonates with a confirmed diagnosis for DS by karyotype analysis (antenatal or postnatal) were included. To ensure homogeneity, neonates with DS due to mosaicism (*n* = 7) or translocation (*n* = 3) were excluded. Since neonatal mortality could not be calculated with this present cohort of newborns with DS that survived the neonatal period and were followed up at the outpatient clinic, a second database was used, called the Dutch national birth registry (Perined)*.* This database comprises data on pregnancies, births, and neonatal outcomes in the Netherlands.

### Outcome

We reviewed the medical files of the 253 neonates with DS for maternal and neonatal characteristics and DS-associated morbidities present during the first 28 days of life, including CHD, gastro-intestinal defects, and persistent pulmonary hypertension of the neonate (PPHN) [9]. The diagnosis of CHD and PPHN was based on a clinical assessment and echocardiography in the first weeks of life performed by a paediatric cardiologist. Patent ductus arteriosus (PDA) was counted as CHD if present as a single heart defect, in infants with gestational age ≥ 37 weeks, and in the absence of PPHN. Persistent foramen ovale (PFO) was not considered as CHD. PPHN was diagnosed by the paediatric cardiologist using common ultrasound signs matching with pulmonary pressures exceeding systemic pressures, like the presence of a right-to-left shunt across the ductus arteriosus (if patent) or signs of suprasystemic right ventricular pressure based on tricuspid regurgitation velocity or systolic movement of the interventricular septum towards the left ventricle.

Data from Perined is used to calculate neonatal mortality (death in the first 28 days of life). Registration at Perined is mandatory for all neonatal intensive care units (NICUs) in the Netherlands. Since critically ill neonates will always be admitted to a NICU in the Dutch health care system, we used the mortality numbers of neonates with DS from Perined as the total mortality number of neonates with DS. Registration at Perined of neonates with DS *without* a NICU indication is done on a voluntary basis. For this reason, it is uncertain if the numbers of total livebirths with DS reported by Perined are complete. Therefore, neonatal mortality was not only calculated using total numbers of DS livebirths extracted from Perined, but also the total numbers of DS livebirths calculated by de Groot et al. [6].

### Statistical analysis

In this retrospective cohort study, we collected data from children with DS that went to outpatient clinics. In the available data, a part of maternal and pregnancy characteristics were missing. Information concerning suspicion/detection of DS during pregnancy and time till postnatal diagnosis was missing in approximately 2%. Information on birthweight was missing in 9%, while details concerning maternal age at birth, parity, Apgar, and the use of prenatal screening in case a child was postnatal diagnosed with DS were missing in 20–30% of cases. Since we did not perform statistical analyses with this data, we reported these characteristics without adjustments for missing data. Because information on cardiac and gastro-intestinal malformations was missing in two cases, respectively one case, no imputation for missing data was done.

As we expected morbidity numbers to be low and privacy reasons did not allow us to obtain the date of birth in our cohort, we provided summarized descriptive statistics and interpreted the results in the context of other published studies. Weijerman et al. reported the prevalence of CHD and PPHN in children born in The Netherlands with DS between 2003 and 2006 [10]. To identify differences between types of cardiac defects over time, we calculated odds ratio and 95% confidence intervals (CI) for the prevalence of cardiac defects in our cohort compared to the cohort of Weijerman et al. [10].

### Ethics approval

The study was approved by the Ethics Committee of Amsterdam University Medical Centers (No. 2018.179) and the Local Research Ethics Committee.

## Results

### Maternal and neonatal characteristics

The mean age of mothers was 34.5 years (*SD* = 5). Among the mothers with a child with DS, 92% had a second-trimester foetal anomaly scan, and 43 of the 186 mothers (23.1%) opted for NIPT and/or FCT. In 65 (26.2%) infants, there was prenatal suspicion of DS. DS was most often suspected after an abnormal 20-week foetal anomaly scan. In 21 (32.3%) of these cases, DS was confirmed with invasive testing. This means that 21 of the 248 neonates (8.5%) were diagnosed with DS before birth. The children were born after a mean gestational age at delivery of 37 weeks (*SD* = 2) with a mean birthweight of 2863 g (*SD* = 639). More than half of the neonates with DS were male (61.7%). Most children were clinically diagnosed with DS on the first day after birth (Table 1).

**Table 1:** Maternal and neonatal characteristics in Down syndrome, *n* = 253

**Characteristics**	**Study cohort**
Mean maternal age, year [mean (*SD*; *N*)]	34.5 (5; 181)
Parity [% (*n*/*N*)] Primipara Multipara	35.9 (71/198) 64.1 (127/198)
Gestational age at delivery, weeks [mean (*SD*; *N*)]	37 (2; 253)
Birthweight, gram [mean (*SD*; *N*)]	2863 (639;233)^a^
Male sex [% (*n*/*N*)]	61.7 (156/253)
Prenatal suspicion of DS [% (*n*/*N*)] Second-trimester foetal anomaly scan Second-trimester foetal anomaly scan + FCT Second-trimester foetal anomaly scan + NIPT Second-trimester foetal anomaly scan + FCT + NIPT Confirmed diagnosis with CVS or AC	26.2 (65/248) 67.7 (44/65) 18.5 (12/65)^b^ 7.7 (5/65) 6.2 (4/65)^b^ 32.3 (21/65)
Postnatal suspicion of DS [% (*n*/*N*)] No prenatal aneuploidy screening Prenatal aneuploidy screening Second-trimester foetal anomaly scan Second-trimester foetal anomaly scan + FCT Second-trimester foetal anomaly scan + NIPT Second-trimester foetal anomaly scan + FCT + NIPT	73.8 (183/248) 12.4 (15/121) 87.6 (106/121) 79.2 (84/106) 18.9 (20/106) 0.9 (1/106) 0.9 (1/106)^c^
Time of clinical diagnosis of DS [% (*n*/*N*)] 1 day 2–7 days ≥ 7 days	83.9% (151/180) 12.2% (22/180) 3.9% (7/180)
Apgar ≤ 5 by 5 min [% (*n*/*N*)] Apgar ≤ 7 by 5 min [% (*n*/*N*)]	4.1 (8/193) 11.4 (22/193)
Number of foetuses involved in a twin pregnancy [% (*n*/*N*)] ^d^	5.2 (13/252)
Neonatal mortality [% (*n*/*N*)]^e^	2.2 (32/1424)

Total numbers varied due to incomplete data collection

*SD* standard deviation, *n* number, *N* total number, *DS* Down syndrome, *FCT* first combined screening test, *NIPT* non-invasive prenatal testing, *CVS* chorionic villus sampling, *AC* amniocentesis

^a^For Dutch population, mean birthweight is circa 3434 g. Birthweight in study population < p 3 in 14.4%; > p 97 in 3.1%

^b^In five cases, the second-trimester foetal anomaly scan showed no abnormalities; in one case, the FCT showed no increased risk for DS

^c^Twin pregnancy

^d^In 13 pregnancies, one of the two children had DS

^e^Based on numbers of deaths registered by Perined *(unpublished)* and total DS livebirths of de Groot et al., 2021

### Neonatal mortality

Neonatal mortality in DS based on Perined data ranged from 2.3 to 6.8%. There was no clear trend visible over time. Neonatal mortality in DS based on total DS livebirths in the Netherlands published by de Groot et al. [6] was lower, ranging from 1.4 to 3.6% (Table 2). Again, no clear trend was seen over time.

**Table 2:** Mortality^a^ in neonates with Down syndrome

	2012	2013	2014	2015	2016	2017	2018
Neonatal deaths in DS (*n*)^b^	5	4	7	3	3	4	6
DS LBs (*n*)^b^	159	171	127	127	131	116	88
Neonatal mortality in DS (%)^b^	3.1	2.3	5.5	2.4	2.3	3.4	6.8
DS LBs (*n*)^c^	233	229	197	195	214	189	167
Neonatal mortality in DS (%)^d^	2.1	1.7	3.6	1.5	1.4	2.1	3.6

*DS* Down syndrome, *LBs* livebirths

^a^Neonatal mortality (%) = number of neonatal deaths in DS LBs/ total DS livebirths × 100%

^b^Based on data of PERINED (*unpublished*)

^c^Based on data of de Groot et al. (2021)

^d^Neonatal deaths in DS based on data of PERINED with DS LBs based on data of de Groot et al. (2021)

### Neonatal morbidity

A CHD was found in 138 of the 251 DS children (55.0%). Atrial septal defect (ASD), atrioventricular septal defect (AVSD), and ventricular septal defect (VSD) were the most common cardiac anomalies (Table 3).

**Table 3:** Congenital heart defects in neonates with Down syndrome born in 2012–2018, *n* = 251^a^

Congenital heart defect	*n*
No	113
Yes	138
Types of congenital heart defect (single and combination of defects)	n (%)^b^
ASD	48 (34.8)
AVSD	48 (34.8)
VSD	47 (34.1)
PDA	10 (7.2)
TOF	6 (4.3)
Other	8 (5.6)
Single defects	105 (76.1)
AVSD	39 (27.5)
VSD	25 (17.6)
ASD	27 (19)
PDA	14 (9.9)
Other	4 (2.8)
Artery lusoria	1
PS	3
Combination of defects	33 (23.9)
AVSD + TOF	1 (0.7)
AVSD + ASD	3 (2.1)
AVSD + VSD	2 (1.4)
AVSD + VSD + ASD	2 (1.4)
VSD + ASD	16 (11.3)
VSD + overriding aorta	1 (0.7)
TOF	5 (3.5)
Other	3 (2.1)
Artery lusoria + bicuspid aortic valve	1
VSD + hypoplastic aortic arch	1
AVSD + ASD + artery lusoria	1

*n* number, *ASD* atrial septal defect, *AVSD* atrioventricular septal defect, *VSD* ventricle septal defect, *PDA* patent ductus arteriosus, *TOF* tetralogy of Fallot, *PS*, pulmonary stenosis

^a^2 cases missing

^b^ Total is > 100% because combinations of CHDs occur

There was a 1.62 (95% CI 1.19, 2.21) higher odds to have a CHD in the cohort of 2012–2018, compared to the cohort DS children born between 2003 and 2006 (43%) [10]. Also, the odds for an ASD was 3.02 higher (95% CI 1.90, 4.81) in the cohort of 2012–2018. No higher odds were found in the other types of CHD. Also, the proportion of complex cardiac defects (AVSD, aortic arch abnormalities, tetralogy of Fallot (TOF), transposition of the great arteries, and single ventricle hearts) was similar in our cohort (22.7%) compared to the cohort of 2003–2006 (25.3%) [10] (Table 4). PPHN was reported in 31 (12.4%) infants with DS. CHD was present in 21 (67.7%) of these infants, which were mainly septal defects. (Table 5). Gastro-intestinal defects were present in 22 DS infants (8.7%), with duodenal atresia being the most often reported anomaly. Vision and hearing disorders, hyperbilirubinemia, and blood cell abnormalities were the most common other comorbidities (Table 6).

**Table 4:** Congenital heart defects in neonates with Down syndrome born in 2012–2018, *n* = 251^a^ compared to 2003–2006, *n* = 482^b^

**Congenital heart defect**	**2012–2018**^a^ ***n* (%)**	**2003–2006**^b^ ***n* (%)**	**OR (95% CI)**
No	113 (45.0)	275 (57.0)	
Yes	138 (55.0)	207 (43.0)	1.62 (1.19, 2.21)
ASD	48 (19.1)	35 (7.3)	3.02 (1.90, 4.81)
AVSD	48 (19.1)	104 (21.6)	0.86 (0.59, 1.26)
VSD	47 (18.7)	69 (14.3)	1.38 (0.92, 2.07)
PDA	10 (4.0)	12 (2.5)	1.63 (0.69, 3.82)
TOF	6 (2.4)	11 (2.3)	1.05 (0.38, 2.87)
Other	8 (3.2)	19 (4.0)	0.80 (0.35, 1.86)
Complex cardiac heart defect^c^	57 (22.7)	122 (25.3)	0.87 (0.61, 1.24)

*n* number, *CI* confidence intervals, *OR* odds ratio, *ASD* atrial septal defect, *AVSD* atrioventricular septal defect, *VSD* ventricle septal defect, *PDA* patent ductus arteriosus, *TOF* tetralogy of Fallot, PS pulmonary stenosis

^a^2 cases missing

^b^Weijerman et al. (2010)

^c^Complex cardiac heart defect: AVSD, aortic arch abnormalities, TOF, transposition of the great arteries and single ventricle hearts

**Table 5:** Neonates with Down syndrome and persistent pulmonary hypertension and the distribution of a concomitant congenital heart defect, *n* = 31

Congenital heart defect (CHD)	*n* (%)
AVSD	6 (19.4)
VSD	5 (16.1)
ASD	4 (12.9)
PS	1 (3.2)
ASD + VSD	5 (16.1)
No CHD	10 (32.3)
Total	31 (100)

*n* number, *ASD* atrial septal defect, *AVSD* atrioventricular septal defect, *VSD* ventricle septal defect, *PDA* patent ductus arteriosus, *TOF* tetralogy of Fallot

**Table 6:** Gastro-intestinal defects and other comorbidity in neonates with Down syndrome, *n* = 252^a^

Congenital gastro-intestinal defect	*n* (%)
No	230 (91.3)
Yes	22 (8.7)
Types of gastro-intestinal defect	
Duodenal atresia Duodenal atresia/web/stenosis Duodenal atresia with pancreas annulare	10 (4.0) 9 1
Hirschsprung’s disease	5 (2.0)
Oesophagus atresia	3 (1.2)
Ileal atresia	1 (0.4)
Anal atresia	1 (0.4)
Duplication cyst Ileum Duodenum	2 (0.8) 1 1
Other comorbidity	*n* (%)
Thrombocytopenia	25 (9.9)
Polycythaemia	23 (9.1)
Hyperbilirubinemia	29 (11.5)
Transient myeloproliferative disorder	10 (4.0)
Hypothyroidism	19 (7.5)
Laryngotracheobronchomalacia	10 (4.0)
Nasal septal anomaly	2 (0.8)
Orofacial cleft	3 (1.2)
Omphalocele	1 (0.4)
Cryptorchidism	7 (2.8)
Vision disorder^b^	31 (14.9)
Hearing disorder^b^	48 (23.1)

*n* number

^a^1 case missing

^b^ For vision and hearing disorders total *N* = 208, 45 cases missing

## Discussion

Our results show a stable neonatal mortality and morbidity in DS, in an era in which uptake of prenatal aneuploidy screening increased and foetal anomaly scans were commonly used.

The neonatal mortality in DS found in this study ranged from 1.4 to 3.6%. This is in line with findings from previously published Dutch studies reporting neonatal mortality in 2003 (1.65%) [2] and 2003–2006 (3.3%) [10]. Other countries found neonatal mortality ranging from 3.7–13% [11–13], where the higher mortality from the USA may be biased by including only neonates with DS that were transferred for specialized care to a children’s hospital [12].

The mortality rate in our cohort was low and seems stable throughout the study period 2012–2018. These findings suggest that an increasing uptake of prenatal aneuploidy screening did not impact neonatal mortality in DS. Still, neonatal mortality is much higher compared to 0.2% in the general population [14]. Only one study, from Hong Kong, described the impact of prenatal aneuploidy screening on neonatal mortality in DS. They found an improvement in neonatal mortality in DS between 1994 and 2014 and a decrease in DS livebirth prevalence. Better treatment of comorbidities, along with a prenatal screening program resulting in more selective abortion of foetuses with DS with more severe congenital abnormalities, may have caused this improvement of neonatal mortality in Hong Kong [13]. The higher a priori risk in 1994 may explain that our colleagues in Hong Kong found a decreased mortality over time, while we described stable mortality since 2003.

When calculating neonatal mortality, it is imperative to have accurate data on the total number of DS livebirths. As livebirths may be underreported in the Perined database, we used an alternative method based on the data reported by de Groot-van der Mooren et al. in 2021 [6]. In that study, the total number of DS livebirths was estimated by adding livebirths with *prenatal* diagnosis to livebirths with *postnatal* diagnosis. The livebirths with *postnatal* diagnosis of DS were collected from all Dutch cytogenetic centres. As expected, this second analysis showed a higher number of DS livebirths and a lower percentage of mortality. Hence, neonatal mortality will be overestimated if based on Perined data exclusively, due to incomplete numbers of DS livebirths.

The prevalence of heart defects among neonates with DS in our cohort is 55%. This is higher than 43% found in a previous Dutch study by Weijerman et al., including DS neonates born in the period 2003–2006 [10]. This higher prevalence of CHD in our study may be partly explained by the fact that, in contrast to the study by Weijerman et al., we included an ASD secundum (that usually arises from an enlarged OFO) as a CHD [10]. Furthermore, improvements in echocardiographic technology over time may have resulted in ascertainment bias, increasing the likelihood of infants being diagnosed with a mild CHD (like ASD).

Of all diagnosed CHDs in our study, 34.8% was an AVSD. This is lower than 54% found by Weijerman et al. [10] However, the proportion of all live born neonates with DS having an AVSD showed only a small difference between our cohort and Weijerman et al. (19.1% versus 21.6%, see Table 4). It seems that the decrease in proportion of AVSD can be explained by the higher overall prevalence of heart defects diagnosed. Furthermore, no clear differences were found in the prevalence of other cardiac defects. These findings implicate that an increasing uptake of prenatal aneuploidy screening caused no shift in type of CHD in DS.

This assumption is supported by data from a large European network of population-based registries for the epidemiologic surveillance of congenital anomalies (EUROCAT). They showed a constant prevalence of congenital cardiac anomalies in neonates with DS (livebirths and foetal deaths from 20 weeks of gestation, excluding termination of pregnancy for foetal anomaly) during 2000–2010. Furthermore, a constant prevalence of ASD and VSD (reported as a less severe cardiac anomaly) was observed. This indicates that the constant prevalence of cardiac anomalies in neonates with DS was due to stable severe *and* less severe cardiac anomalies [15].

Our findings are also in line with the study from the USA among 9122 neonates with DS in which a complex CHD was found in 6% (including single ventricle disease and other cardiac defects commonly requiring surgical repair), an AVSD in 22%, a VSD in 22%, and a TOF in 4% of neonates with DS. No differences were observed in the pre-NIPT era (2007–2010) versus post-NIPT era (2014–2018) [16].

It is important to acknowledge that some reports *do* suggest a shift in type of CHD. For example, in Sweden complex congenital heart defect (including AVSD, aortic arch abnormalities, TOF, transposition of the great arteries, and single ventricle hearts) have become less common in neonates with DS between 1992 and 2012 [17]. Similarly in Germany, the number of AVSDs in neonates with DS was reduced by more than 14% from the period of 2005–2009 to 2010–2014 [18].

This inconsistency is also reported in a literature review of 56 articles showing that assessing trends in total prevalence and types of CHD in DS was difficult. While some studies indicate a trend towards a milder phenotype, others do not. The lack of standard nomenclature for CHD impeded comparison between the studies [19].

Besides CHD, the most often reported congenital malformation in DS is a gastro-intestinal defect. In our study, a prevalence of gastro-intestinal defects of 8.7% was found. Duodenal atresia was most often reported (45.5%). In literature, percentages of gastro-intestinal defects in DS ranged from 3 to 13%, with duodenal atresia as the most frequently reported defect varying from 39 to 67% [15, 20]. The broad range can be explained by the variation of study population (livebirths, stillbirths, foetuses). This makes comparison between studies difficult, although it seems that over the last decades the prevalence of gastro-intestinal defects has remained stable [15, 20].

The findings of the current study do not support our hypothesis that the introduction of prenatal aneuploidy screening programs have resulted in a decreasing neonatal mortality and a more favourable phenotype in live born neonates with DS. Several developments seem to play a role. First, a proportion of women refrain from prenatal aneuploidy screening [21, 22]. An important reason for declining the offer of prenatal aneuploidy screening are value related (i.e. not considering DS a condition severe enough to justify termination of pregnancy) [21, 23]. Second, prenatal aneuploidy screening can also be used to prepare for the birth of a child with DS. Third, an increasing uptake of prenatal aneuploidy screening was mainly seen in younger mothers, with a lower consequent impact on the number of prenatal diagnoses of DS.

Because nowadays most cardiac and gastro-intestinal defects are treatable [10, 24], it may imply that for some parents, the cardiac or gastro-intestinal malformations that might have been suspected on foetal anomaly scan do not conflict with their decision to decline prenatal aneuploidy screening or accept screening and continue the pregnancy in case of DS.

Whereas stable neonatal mortality and morbidity were found, the incidence of PPHN in neonates with DS increased (12.4%). In a previous cohort, PPHN occurred in 5.2%, and in the general population an incidence of 0.1% is reported [10]. Additionally, in other countries a high incidence of PPHN was found, ranging from 17 to 35% [9, 25]. This can be explained by the development of a more systematic approach to the diagnosis of PPHN using objective echocardiography techniques and differences in diagnostic criteria. These findings underline the need for awareness of PPHN in neonates with DS, in particular for the Dutch perinatal care setting, where there is a high number of home births and early postnatal discharge from hospital after uncomplicated deliveries. In these cases, a simple pulse oximetry may be a safe and reliable method to measure the peripheral oxygen saturation and detect (subclinical) cyanosis, which may be an indication for PPHN [26].

There are some limitations to this study which can lead to selection bias. First our cohort does not involve all DS livebirths in the Netherlands but includes patients of the outpatient clinics of five hospitals—one academic hospital and four general hospitals. Complex CHDs which are prenatally detected and need surgical treatment directly after birth may have been transported to a different academic hospital with expertise in cardiac surgery. As a result complex CHD may be underestimated. The follow-up of most of these patients however is in the general hospital nearby the residence of the child (shared care), but there may be exceptions.

Second, only neonates seen in outpatient clinics are included and therefore neonates that died were excluded. This may have caused an underestimation of complex cardiac defects or other major anomalies. However, research has shown that over the last decades, neonatal mortality in DS is becoming less dependent on CHDs and is more often caused by other neonatal pathology [10]. Thirdly, in the Netherlands regional differences in prenatal screening uptake are observed. First tier NIPT uptake ranged from 31.8% in the northeast to 67.9% in the northwest and center of the Netherlands [5]. This means that in our cohort (from northwest and center), the uptake may be higher than in a national cohort. However, if an increase in uptake of prenatal screening gives a decrease on the prevalence of morbidity and mortality (our hypothesis), we would expect that with our study the chance to confirm our hypothesis is enlarged.

Our findings suggest that increasing uptake of prenatal screening does not necessarily change mortality or morbidity in DS. Therefore, up-to-date knowledge concerning the prevalence, treatment, and prognosis of congenital malformations associated with DS is necessary to maintain high quality care for DS children. Additionally, this study increases awareness for PPHN in neonates with DS. Further improvement in the national registry of perinatal characteristics in DS is essential for updating national prenatal aneuploidy screening programs and clinical guidelines. Besides the use of consistent nomenclature, other factors which impact the prevalence and severity of a congenital malformation should be analysed [17].

In conclusion, this study indicates that although the uptake of prenatal aneuploidy screening increased, neonatal mortality and morbidity in DS appears to be stable. An increased incidence of PPHN was found.

## Data Availability

The data that support the findings of this study are available from the corresponding author upon reasonable request.

## Abbreviations

AC: Amniocentesis
ASD: Atrial septal defect
AVSD: Atrioventricular septal defect
CHD: Congenital heart disease
CI: Confidence intervals
CVS: Chorionic villus sampling
DS: Down syndrome
EUROCAT: Epidemiologic surveillance of congenital anomalies
FCT: First combined screening test
LB: Livebirth
NICU: Neonatal intensive care unit
NIPT: Non-invasive prenatal testing
*n*: Number
*N*: Total number
OR: Odds ratio
PDA: Patent ductus arteriosus
PFO: Persistent foramen ovale
PPHN: Persistent pulmonary hypertension of the neonate
PS: Pulmonary stenosis
SD: Standard deviation
TOF: Tetralogy of Fallot
VSD: Ventricular septal defect

## References

[1] Bull MJ (2020). Down syndrome. N Engl J Med.

[2] Weijerman ME, van Furth AM, Vonk Noordegraaf A, van Wouwe JP, Broers CJ, Gemke RJ (2008). Prevalence, neonatal characteristics, and first-year mortality of Down syndrome: a national study. J Pediatr.

[3] Breveglieri G, D'Aversa E, Finotti A, Borgatti M (2019). Non-invasive prenatal testing using fetal DNA. Mol Diagn Ther.

[4] Committee Opinion No. 640 (2015) Cell-free DNA screening for fetal aneuploidy. Obstet Gynecol 126:e31–e37. 10.1097/AOG.0000000000001051 10.1097/AOG.0000000000001051 26287791

[5] van der Meij KRM, de Groot-van MM, Carbo EWS, Pieters MJ, Rodenburg W, Sistermans EA, Cornel MC, Henneman L, Dutch NC (2021). Uptake of fetal aneuploidy screening after the introduction of the non-invasive prenatal test: a national population-based register study. Acta Obstet Gynecol Scand.

[6] de Groot-van der Mooren M, de Graaf G, Weijerman ME, Hoffer MJV, Knijnenburg J, van der Kevie-Kersemaekers AMF, Kooper AJA, Voorhoeve E, Sikkema-Raddatz B, van Zutven L, Srebniak MI, Huijsdens-van Amsterdam K, Engelen JJM, Smeets D, van Kaam AH, Cornel MC (2021). Does non-invasive prenatal testing affect the livebirth prevalence of Down syndrome in the Netherlands? A population-based register study. Prenat Diagn.

[7] Natoli JL, Ackerman DL, McDermott S, Edwards JG (2012) Prenatal diagnosis of Down syndrome: a systematic review of termination rates (1995–2011). Prenat Diagn 32:142–153. 10.1002/pd.2910 10.1002/pd.2910 22418958

[8] Choudhry MS, Rahman N, Boyd P, Lakhoo K (2009). Duodenal atresia: associated anomalies, prenatal diagnosis and outcome. Pediatr Surg Int.

[9] Shah PS, Hellmann J, Adatia I (2004). Clinical characteristics and follow up of Down syndrome infants without congenital heart disease who presented with persistent pulmonary hypertension of newborn. J Perinat Med.

[10] Weijerman ME, van Furth AM, van der Mooren MD, van Weissenbruch MM, Rammeloo L, Broers CJ, Gemke RJ (2010). Prevalence of congenital heart defects and persistent pulmonary hypertension of the neonate with Down syndrome. Eur J Pediatr.

[11] Ergaz-Shaltiel Z, Engel O, Erlichman I, Naveh Y, Schimmel MS, Tenenbaum A (2017). Neonatal characteristics and perinatal complications in neonates with Down syndrome. Am J Med Genet A.

[12] Cua CL, Haque U, Santoro S, Nicholson L, Backes CH (2017). Differences in mortality characteristics in neonates with Down’s syndrome. J Perinatol.

[13] Chua GT, Tung KTS, Wong ICK, Lum TYS, Wong WHS, Chow CB, Ho FK, Wong RS, Ip P (2020). Mortality among children with down syndrome in Hong Kong: a population-based cohort study from birth. J Pediatr.

[14] https://www.peristat.nl/ . Accessed 1 Nov 2021

[15] Morris JK, Garne E, Wellesley D, Addor MC, Arriola L, Barisic I, Beres J (2014). Major congenital anomalies in babies born with Down syndrome: a EUROCAT population-based registry study. Am J Med Genet A.

[16] Hart SA, Nandi D, Backes CH, Cua CL (2021). Impact of prenatal screening on congenital heart defects in neonates with Down syndrome in the US. Pediatr Res.

[17] Bergstrom S, Carr H, Petersson G, Stephansson O, Bonamy AK, Dahlstrom A, Halvorsen CP, Johansson S (2016). Trends in congenital heart defects in infants with Down syndrome. Pediatrics.

[18] Pfitzer C, Helm PC, Rosenthal LM, Berger F, Bauer UMM, Schmitt KR (2018). Dynamics in prevalence of Down syndrome in children with congenital heart disease. Eur J Pediatr.

[19] Santoro SL, Steffensen EH (2021). Congenital heart disease in Down syndrome – a review of temporal changes. J Congenit Heart Dis.

[20] Stoll C, Dott B, Alembik Y, Roth MP (2015). Associated congenital anomalies among cases with Down syndrome. Eur J Med Genet.

[21] Crombag NM, Page-Christiaens GC, Skotko BG, de Graaf G (2020). Receiving the news of Down syndrome in the era of prenatal testing. Am J Med Genet A.

[22] van Schendel RV, Dondorp WJ, Timmermans DR, van Hugte EJ, de Boer A, Pajkrt E, Lachmeijer AM, Henneman L (2015). NIPT-based screening for Down syndrome and beyond: what do pregnant women think?. Prenat Diagn.

[23] Crombag NMTH, van Schendel RV, Schielen PCJI, Bensing JM, Henneman L (2016) Present to future: what the reasons for declining first-trimester combined testing tell us about accepting or declining cell-free DNA testing. Prenatal Diag 36:587–590. 10.1002/pd.4824 10.1002/pd.4824 27061402

[24] Bethell GS, Long AM, Knight M, Hall NJ, Baps C (2020). The impact of trisomy 21 on epidemiology, management, and outcomes of congenital duodenal obstruction: a population-based study. Pediatr Surg Int.

[25] Martin T, Smith A, Breatnach CR, Kent E, Shanahan I, Boyle M, Levy PT, Franklin O, El-Khuffash A (2018). Infants born with Down syndrome: burden of disease in the early neonatal period. J Pediatr.

[26] Narayen IC, Blom NA, Bourgonje MS, Haak MC, Smit M, Posthumus F, van den Broek AJ, Havers HM, te Pas AB (2016). Pulse oximetry screening for critical congenital heart disease after home birth and early discharge. J Pediatr.

